# Efficacy of desensitizing products containing 8% arginine and calcium carbonate for hypersensitivity relief in MIH-affected molars: an 8-week clinical study

**DOI:** 10.1007/s00784-016-2024-8

**Published:** 2016-12-21

**Authors:** Katrin Bekes, Karolin Heinzelmann, Stefan Lettner, Hans-Günter Schaller

**Affiliations:** 1 School of Dentistry, Department of Paediatric Dentistry, Medical University Vienna, Sensengasse 2a, 1090 Vienna, Austria; 2 Department of Operative Dentistry and Periodontology, Department of Paediatric Dentistry, Martin-Luther-University Halle-Wittenberg, University School of Dental Medicine, Gr. Steinstr. 19, 06108 Halle, Germany; 3 Karl Donath Laboratory for Hard Tissue and Biomaterial Research, Statistics, Medical University Vienna, Sensengasse 2a, 1090 Vienna, Austria

**Keywords:** Molar incisor hypomineralization (MIH), Hypersensitivity, Desensitizing, Arginine, Children

## Abstract

**Objectives:**

The objective of this study was to compare the efficacy in reducing hypersensitivity in molar incisor hypomineralization (MIH)-affected molars immediately and over 8 weeks combining a single in-office application and a homed-based program with desensitizing products containing 8% arginine and calcium carbonate.

**Materials and methods:**

Nineteen children with at least one MIH-affected molar with hypersensitivity were included. Hypersensitivity was assessed with an evaporative (air) stimulus and a tactile stimulus. Each child received a single in-office treatment with a desensitizing paste containing 8% arginine and calcium carbonate (elmex Sensitive Professional desensitizing paste), followed by 8 weeks of brushing twice daily with a desensitizing toothpaste containing 8% arginine, calcium carbonate with 1450 ppm fluoride (elmex Sensitive Professional toothpaste), using the elmex Sensitive Professional toothbrush. Additionally, the corresponding mouthwash (elmex Sensitive Professional mouthwash) was used. Clinical assessments were made at baseline, immediately after the in-office treatment and after 1, 2, 4 and 8 weeks of brushing twice daily.

**Results:**

Fifty-six molars with an air blast hypersensitivity score of 2 or 3 (Schiff Cold Air Sensitivity Scale) were included. Application of the desensitizing paste decreased hypersensitivity significantly immediately and throughout the 8 weeks recalls (*p* < 0.001).

**Conclusions:**

In conclusion, 8% arginine and calcium carbonate were able to reduce hypersensitivity successfully during this 8-week trial.

**Clinical relevance:**

Hypersensitivity is a major complaint in patients with MIH. This is the first study evaluating the desensitizing effect of a desensitizing paste containing 8% arginine and calcium carbonate in patients with MIH.

## Introduction

The term molar incisor hypomineralization (MIH) was introduced in 2001 to describe the clinical picture of enamel hypomineralization of systemic origin affecting one or more first permanent molars that are associated frequently with affected incisors [1]. Its etiology remains unknown [2]. It has been suggested that MIH is a genetic condition based on its prevalence [3] and additionally, that childhood illness is likely to be associated with MIH [4].

Patients affected by MIH present several clinical problems, including rapid wear, enamel loss, increased susceptibility to caries, loss of fillings, and most of all, severe hypersensitivity often resulting in severe discomfort [5]. Children often report that hot and cold or sweet drinks and meals, toothbrushing, and even air flow cause sensitivity [1, 6, 7]. At dental examination, behavior management problems and even dental fear are common [5]. The reason for hypersensitivity, however, is still not fully understood [8].

At present, the preventive approach includes thorough oral hygiene with fluoride toothpaste as well as the application of other topical fluoride varnishes [5]. Casein phosphopeptide-amorphous calcium phosphate (CPP-ACP) oral care products are similarly recommended for remineralization and desensitization [7, 9]. The CPP-ACP can interact with fluoride ions, producing an amorphous calcium phosphate stabilized by CPP at the tooth surface and providing soluble calcium, fluoride, and phosphate ions to promote remineralization with fluorapatite that is more acid resistant [10]. Although there is no evidence at present to support treating MIH-affected teeth, all these products are empirically proven to seal, desensitize, and enhance mineralization of the hypomineralized areas [5, 7].

Recently, a new technology based on arginine has been launched (ProArgin™). The ProArgin™ desensitizing paste contains hydrated silica, calcium carbonate, glycerin, 8% arginine, water, bicarbonate, cellulose gum, and sodium saccharin. It has been found to be effective in reducing dentin hypersensitivity as a self-applied or professionally applied agent [11]. In the case of dentin hypersensitivity, it is instant and lasts up to 8 weeks [12, 13].

The present trial was undertaken to evaluate the clinical efficacy of desensitizing products containing 8% arginine and calcium carbonate (ProArgin™) in relieving hypersensitivity in MIH molars immediately and over 8 weeks days following a single topical application in-office in combination with an at home treatment.

The hypothesis tested in the present paper was that a combined use of ProArgin™ minimizes the hypersensitivity in affected molars.

## Materials and methods

### Study design and population

This trial was designed as a nonrandomized, single group study, to evaluate the effect of an arginine-containing desensitizing paste (Elmex Sensitive Professional desensitizing paste, CP Gaba GmbH, Germany) on desensitization of hypersensitive molars with MIH. For ethical reasons, the study did not include a placebo (control) group. Approval for this clinical investigation was obtained from the ethics committee of the local University Review Board (Martin-Luther-University Halle-Wittenberg; Approval: 2014–34). Written statements of consent were read and signed by parents and children prior to their participation in the study.

Patients for this study were recruited from the Department of Paediatric Dentistry, Martin-Luther-University Halle-Wittenberg, Halle (Saale), Germany. The criteria proposed by the European Academy of Paediatric Dentistry (EAPD) [14] were used for the diagnosis of MIH, which includes the presence of demarcated opacities, post-eruptive enamel breakdown, atypical restorations, and extraction due to MIH in at least one first permanent molar. Demarcated opacities with a diameter of <1 mm were not considered in the analysis.

The inclusion criteria were children and adolescents aged 6–14, at least one hypersensitive molar with MIH which had a qualifying response to air blast stimuli applied for 1 s as defined by a score of 2 or 3 on the Schiff Cold Air Sensitivity Scale (SCASS), and signed informed consent form. Exclusion criteria were systemic diseases, long-term medication, hypomineralized molar due to other medical conditions, hypersensitive study teeth with contributing etiologies other than recognized clinically as being associated with MIH, use of any desensitizing product within the past 6 months, ongoing treatment with antibiotics and/or anti-inflammatory drugs, caries, restorations in study teeth, allergies to arginine, oral care products, test products, personal care consumer products, or their ingredients.

Two proficient dentists examined potential children for inclusion into the trial. Possible MIH-affected molars for inclusion were selected in response to the following two stimuli: air blast hypersensitivity and tactile hypersensitivity. The air was delivered from a standard dental unit air syringe at maximal pressure (45 psi) and at an environmental temperature of 19–24 °C. The air current was applied for 1 s at a distance of 1 cm and perpendicular to the occlusal surface of the tooth. Neighboring teeth were shielded with cotton rolls or with the fingers of the examiner. The SCASS was used to assess subject response to this stimulus. The scale is scored as follows: 0 = subject does not respond to the stimulus; 1 = subject does not respond to the stimulus, but considers stimulus to be painful; 2 = subject responds to air stimulus and moves from the stimulus; and 3 = subject responds to air stimulus, moves from the stimulus, and requests immediate discontinuation of the stimulus [11]. Five minutes later, tactile hypersensitivity was assessed by scratching on the surface of the MIH-affected tooth with a dental explorer (max. twice scratches back and forth). The children scored pain intensity with the Wong Baker Faces Scale (WBFS) (0 = no hurt and 10 = hurts worst) [15].

### Clinical examination

Study subjects were instructed to refrain from all oral hygiene procedures, chewing gum, and pain killers for 8 h and from eating and drinking for 2 h prior to examinations.

The targeted MIH molar was cleaned with a cotton pellet. Then, the operator applied elmex Sensitive Professional desensitizing paste in accordance with the manufacturer’s instructions using a rotary cup filled with the paste. Using a low to moderate speed, the paste was polished into each included tooth. The product was applied for 3 s and then the procedure was repeated.

Moreover, the children were instructed to use the assigned home-use toothpaste (elmex Sensitive Professional tooth paste) and the toothbrush (elmex Sensitive Professional tooth brush) twice per day for at least 2 min using a pea-sized amount for the duration of the study. Additionally, they were briefed to rinse with 20 ml of the corresponding mouthwash (elmex Sensitive Professional mouth wash) for 30 s after tooth brushing. Subjects were instructed to use only their assigned toothpaste, toothbrush, and mouthwash and to discontinue all other oral hygiene practices.

All participants were evaluated immediately after treatment and after 1, 2, 4, and 8 weeks. Tactile and air blast hypersensitivity examinations were performed following the same methodology. The investigator recorded the patients’ scores on a blank form, only indicating the patient number, the numbers of the treated teeth, and a space to note the air-blast and probe-scratch scores. Baseline and follow-up examinations were done by one dentist.

### Sample size

Estimation of sample size was based on findings from a previous study, showing a minimum significant difference of 1 in Schiff scores. With a total of 18 patients entering this study, the probability of the study detecting a treatment difference was 80% at a two-sided 0.05 significance level. The plan was to conservatively enroll at least 19 participants.

### Statistical analysis

For inference, analyses were based on teeth, while still respecting the influence of the patient as the statistically independent unit by using a random effects approach [16]. Further, it was necessary to consider the ordinal nature of the measurements; therefore, cumulative logit random intercept models [17] were fitted using the ordinal package [18]. These models included SCASS and WBFS, respectively, as a dependent variables; time as an independent variable and maxilla/mandible as well as patient level characteristics (gender, age) as covariates; and finally nested random effects for patient ID and tooth. Subjects who dropped out of the study are included in the statistical analysis until the time of dropout. We assumed data to be missing at random [19]. The proportional odds assumption was assessed graphically using partial residual plots. We tested hypotheses about the time effect using Wald statistics and adjusting for multiple testing using the multivariate single-step method [20]. Results are presented as least square means (LS-means) on the log odds scale, including an asymptotic 95% confidence interval. All computations were done using R version 3.3.1 [21], and statistical graphs were created using package ggplot2 [22].

## Results

Nineteen patients (mean age 8.2 years ± 1.9; 45% female) with 56 teeth were included in the study. The characteristics of the sample in terms of baseline variables are shown in Table 1. Sixteen participants (44 teeth) completed all stages of the study, and there were no complaints or reactions associated. Four subjects dropped out, two of them were sisters.

**Table 1:** Characteristics of the MIH-affected molars

MIH molar	Total	Schiff score	
		2 *N* (%)	3 *N* (%)
Total	56	50 (89.3)	6 (10.7)
16	12	12 (100.0)	0 (0.0)
26	13	9 (69.2)	4 (30.8)
36	16	15 (93.8)	1 (6.2)
46	15	14 (93.3)	1 (6.7)

**Fig. 1:**
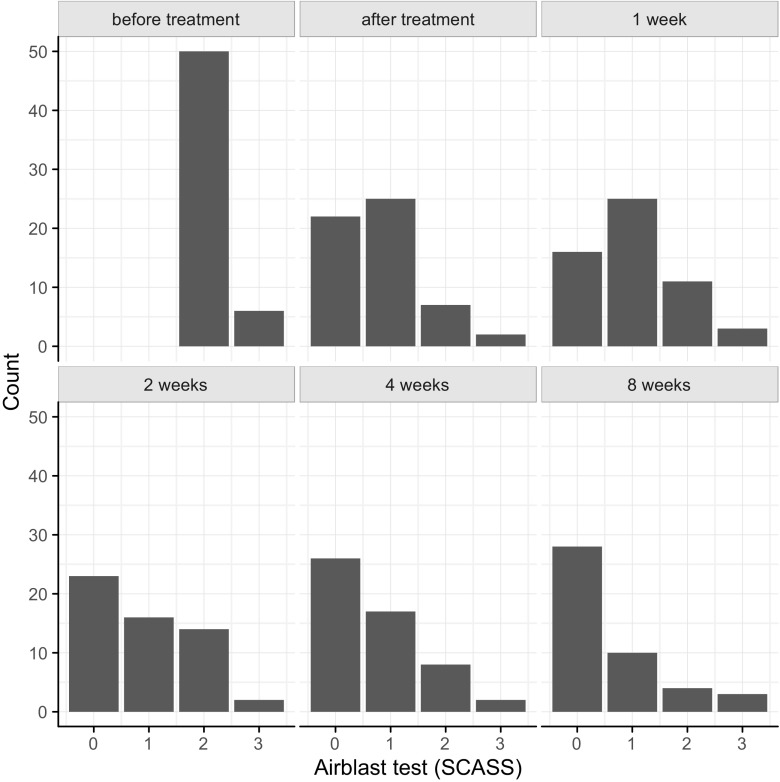
Airblast test scores per tooth for all time points

The mean tactile and air blast hypersensitivity scores measured at the baseline examination, immediate after treatment and after 1, 2, 4, and 8 weeks are shown in Table 2. Airblast test scores per tooth for all time points are presented in Fig. 1.

**Table 2:** Mean scores and standard deviations for the air blast test (Schiff score) and the tactile test (Wong Baker Faces Scale) at different time points

	Mean (SD)
Airblast test	
Before treatment	2.1 (0.3)
Immediately after treatment	0.8 (0.8)
After 1 week	1.0 (0.9)
After 2 weeks	0.9 (0.9)
After 4 weeks	0.7 (0.9)
After 8 weeks	0.8 (0.9)
Tactile test	
Before treatment	2.1 (2.6)
Immediately after treatment	0.8 (1.4)
After 1 week	0.9 (1.4)
After 2 weeks	0.7 (1.0)
After 4 weeks	0.8 (1.4)
After 8 weeks	0.6 (1.1)

*SD* standard deviation

We used a cumulative logit model to evaluate treatment results over time. In this model, we compared all later measurements against one baseline measurement point directly before treatment as well with one baseline measurement point directly after treatment. Estimates from the cumulative logit model on the log-odds scale are displayed in Tables 3 and 4.

**Table 3:** LS-means for air blast and tactile test at different time points for the cumulative logit model

Time	LS-mean	SE	CI_0.025_	CI_0.975_
Airblast test				
Before treatment	2.67	0.53	1.63	3.72
After treatment	−3.14	0.52	−4.16	−2.12
After 1 week	−2.26	0.49	−3.22	−1.30
After 2 weeks	−2.69	0.51	−3.69	−1.70
After 4 weeks	−3.51	0.54	−4.57	−2.45
After 8 weeks	−4.13	0.59	−5.29	−2.97
Tactile test				
Before treatment	−2.66	0.67	−3.97	−1.36
Immediately after treatment	−4.82	0.75	−6.28	−3.35
After 1 week	−4.35	0.72	−5.77	−2.94
After 2 weeks	−4.93	0.75	−6.41	−3.46
After 4 weeks	−4.65	0.74	−6.10	−3.19
After 8 weeks	−5.57	0.80	−7.14	−4.00

*LS* least squares, *SE* standard error, *CI* confidence interval

**Table 4:** Contrasts for air blast and tactile test comparing each time point to the baseline time point (“before treatment”) and the time point directly “after treatment”

Contrast	Estimate	SE	*p* value
Airblast test			
Compared to *before treatment*			
After treatment	−5.82	0.68	<0.001
After 1 week	−4.93	0.64	<0.001
After 2 weeks	−5.37	0.66	<0.001
After 4 weeks	−6.18	0.71	<0.001
After 8 weeks	−6.80	0.76	<0.001
Compared to *after treatment*			
After 1 week	0.88	0.42	0.036
After 2 weeks	0.45	0.42	0.294
After 4 weeks	−0.37	0.44	0.401
After 8 weeks	−0.99	0.49	0.043
Tactile test			
Compared to *before treatment*			
After treatment	−2.15	0.47	<0.001
After 1 week	−1.69	0.45	<0.001
After 2 weeks	−2.27	0.47	<0.001
After 4 weeks	−1.98	0.47	<0.001
After 8 weeks	−2.91	0.53	<0.001
Compared to *after treatment*			
After 1 week	0.46	0.46	0.310
After 2 weeks	−0.12	0.47	0.806
After 4 weeks	0.17	0.48	0.721
After 8 weeks	−0.75	0.52	0.150

Estimates are on the log-odds scale

*SE* standard error

Results from both, tactile and air blast hypersensitivity scores were similar: for air blast hypersensitivity scores, log odds of having higher scores were highest “before treatment” with 2.67, which was significantly higher than at all other time points (*p* < 0.001). While there was a significant increase (0.88, *p* = 0.036) in scores from “after treatment” (−3.14) to “after 1 week” (−2.26), dropping again significantly at 8 weeks (−4.13, *p* = 0.043), essentially Schiff scores stayed at a relatively stable low level compared to “before treatment.”

This levelling off of scores was more pronounced with tactile hypersensitivity scores, where we found again an improvement in scores “before treatment” (−2.15) compared to all other time points (*p* < 0.001), and no significant differences from “after treatment” (−4.82) to later on.

## Discussion

This is the first clinical trial to examine the efficacy of desensitizing products containing 8% arginine and calcium carbonate (ProArgin™) in relieving hypersensitivity in MIH molars immediately and over 8 weeks days following a single topical application in-office in combination with a homed-based treatment. Immediately after treatment as well as after 8 weeks, the desensitizing products were effective in decreasing hypersensitivity.

As air blast and tactile stimuli are the most preferred methods to assess sensitivity in patients with dentin hypersensitivity [13], we adopted this approach for our study. All teeth included in the trial were subjected both stimuli as they are physiological, encountered in everyday life and are easily controlled. When applied to intact teeth, little discomfort results from either of these two stimuli [23]. For evaluation, the visual analogue scale (VAS) is a common method for the quantification of pain severity in adult patients. It is a continuous outcome measure consisting of a scale from 0 to 10 with low and high end points of no pain and worst pain [24]. In contrast, facial expression drawings (“faces scales”) are a popular method of pain severity assessment in pediatric populations [25, 26]. Therefore, the WBFS was used in the present study [15].

At present, the overall preventive approach and advice comprise the use of topical fluoride varnishes in office [5] and 0.4% stannous fluoride gels on a daily basis [27]. Home application of a CPP-ACP-containing cream is also advised to help seal, desensitize, and act as a source of bioavailable calcium and phosphate for the erupting MIH molar [5, 9, 28]. These current treatment regimes are more or less centered around empiricism as there is no research at present to evaluate the efficacy of these products in MIH patients.

Due to this large lack of information and the need for further clinical studies focusing on hypersensitive MIH teeth, the present trial investigated the efficacy of ProArgin™ in reducing hypersensitivity in MIH-affected molars. ProArgin™ was recently developed to treat dentine hypersensitivity [29]. The essential components of this product are arginine (8%), a pH buffer, and calcium carbonate. The technology was introduced originally as SensiStat in the late 1990s; since 2009, it has been known as Colgate Sensitive Pro-Relief Desensitizing Paste [30, 31]. Clinical studies have demonstrated a relieving effect of Pro Argin in tooth sensitivity when professionally applied [11, 32]. As these products are well-known desensitizing agents, however, we aimed to benefit from the desensitizing effects in our patients as well.

Mean discomfort scores reduced for both stimuli at all follow-ups, staying at a much improved level for the duration of the study (8 weeks). While there was another significant improvement in Schiff scores at 8 weeks, we are at this point inclined to think that this may be a random artifact in our data rather than a real long-term drop, especially considering its rather low size.

The potential of the arginine and calcium carbonate-containing formulas to interact with the MIH-affected enamel surface has not been investigated yet. Imaging of dentine surfaces treated with 8% arginine and calcium carbonate compositions reveals surface coverage and coverage of the tubule orifices.

As this is the first clinical trial to examine the development and management of hypersensitivity in MIH patients as well as the first description of a treatment with arginine-containing pastes, no direct comparisons can be made between current and previous data. To our knowledge, only one study exists focusing on hypersensitivity treatment in MIH-affected incisors. Özgul et al. evaluated the effect of desensitizing agents (fluoride, CPP-ACP, and fluoride-containing CPP-ACP) applied with and without ozone to incisors affected by MIH [33]. Treatment and application of the products were performed at baseline and after 4 weeks. All desensitizing methods tested were found to significantly reduce hypersensitivity in teeth with MIH after 3 months of clinical follow-up. Immediately after treatment, CPP-ACP with and without ozone as well as fluoride-containing CPP-ACP groups showed significantly greater reductions in hypersensitivity compared to the fluoride groups. After 4 weeks (immediately after retreatment) and at the end of the study (after 3 months), no statistically significant differences in hypersensitivity reduction were observed between any of the groups.

One of the major limitations of this study is the lack of a negative control, which might have affected the interpretation of our results. However, we considered it unethical to have a negative control. Other limitations include the subjective nature of hypersensitivity assessment and the knowledge of participating in a trial. Compliance bias could not have influenced participant responses as they were not personally related to the investigator nor were they offered any incentive to participate in the trial. We recommend further research to test the efficacy of these desensitizing products, a longer duration of follow-up and the assessment of different grades of sensitivity among a larger sample size to confirm the results of our study. DH is a universal condition, and therefore, the findings of this study can be generalized for all experiencing it.

## Conclusion

The present investigation of patients with hypersensitive MIH molars is the first study evaluating the efficacy of ProArgin™ technology in relieving hypersensitivity. In conclusion, a single topical application of elmex Sensitive Professional desensitizing paste in conjunction with at home-based treatment provided instant significant relief from hypersensitivity in MIH molars. Furthermore, it was effective in maintaining desensitization significantly for 8 weeks.
